# NAD^+^ Homeostasis and Autophagy: Integrated Control Through Nutrient Signaling in Yeast and Mammals

**DOI:** 10.3390/cells14191495

**Published:** 2025-09-24

**Authors:** Matilda McDaniel, Lan-Hsuan Lee, Su-Ju Lin

**Affiliations:** Department of Microbiology and Molecular Genetics, College of Biological Sciences, University of California, Davis, CA 95616, USA; mmcdaniel@ucdavis.edu (M.M.); lhslee@ucdavis.edu (L.-H.L.)

**Keywords:** NAD^+^, autophagy, nutrient sensing, sirtuin

## Abstract

Nicotinamide adenine dinucleotide (NAD^+^) is an essential metabolite facilitating redox and biochemical reactions in many cellular processes. Maintaining NAD^+^ homeostasis is critical for proper cellular function, and abnormalities in NAD^+^ metabolism have been associated with various human diseases. However, the mechanisms underlying its regulation and interconnection with nutrient-sensing pathways remain incompletely understood. Recent studies show that autophagy, a conserved catabolic pathway essential for cellular homeostasis, plays an important role in maintaining the NAD^+^ pool. NAD^+^ may also impact autophagy through its regulation of cellular metabolism and sirtuins, a family of NAD^+^-dependent deacetylases. Given the complexity of these pathways, their mechanistic interconnection is not fully understood. Here, we discuss studies examining the interactions of NAD^+^ metabolism, autophagy, and nutrient-sensing pathways, with a focus on the budding yeast *Saccharomyces cerevisiae* and connections to mammalian systems. We also discuss the role of sirtuins in these pathways and the impacts of NAD^+^ precursor supplementation. This review provides insights into how nutrient-sensing pathways may mediate the co-regulation of NAD^+^, autophagy, and cellular homeostasis. The studies discussed provide the basis for the development of future research directions that may inform potential therapeutic targets for human disorders associated with the dysregulation of NAD^+^ metabolism and autophagy.

## 1. Introduction

Nicotinamide adenine dinucleotide (NAD^+^) is an essential metabolite that plays a key role in cell signaling and energy production. Its oxidized (NAD^+^) and reduced (NADH) forms serve as cofactors in key metabolic pathways, including glycolysis, the tricarboxylic acid (TCA) cycle, and oxidative phosphorylation. Its role in central metabolism permits its use as a signaling molecule to inform the cell about its metabolic state. In addition to its role as a redox cofactor, NAD^+^ is also a substrate for NAD^+^-consuming enzymes, including sirtuins [1,2,3], poly (ADP-ribose) polymerases (PARPs) [4], and the NAD^+^ hydrolases, CD38 (Cluster of Differentiation 38) [5,6] and SARM1 (Sterile Alpha Motif and TIR Motif Containing 1) [7,8]. Therefore, NAD^+^ intersects with and contributes to a network of sensors, transducers, transcription factors, and post-translational modifications (PTMs) that signal the cell of their nutrient state [9,10,11,12,13]. Although major NAD^+^ biosynthetic enzymes have been extensively studied, how NAD^+^ metabolism is regulated and how its regulation connects to other signaling pathways remains unclear. Recent studies have connected NAD^+^ metabolism with autophagy; however, the detailed mechanisms remain to be further studied. Autophagy is a conserved catabolic process that is paramount in maintaining homeostasis through metabolic reprogramming in response to stress and nutrient deprivation [14,15]. The basal rate of autophagy is low, but upregulated in response to pro-autophagy signals, and loss of autophagy machinery is correlated with reduced survival in many systems [16,17]. While most cellular NAD^+^ (and NADH) is bound to proteins, its intermediates may enter the vacuole/lysosome for turnover via autophagy. For example, in yeast, it has been shown that autophagy induced by nitrogen starvation increases the intermediates nicotinamide riboside (NR), nicotinic acid (NA), and nicotinamide (NAM) in an Atg14-dependent manner [18]. In mammalian cells, autophagy is required for proper maintenance of NAD^+^ pools, and its impairment triggers NAD^+^ depletion and cell death [19].

Dysregulation of autophagy and NAD^+^ metabolism has been implicated in aging and age-related diseases, including cancer and neurodegeneration [20,21]. For example, many cancer cells have altered rates of autophagy and higher NAD^+^ turnover than normal, facilitating aberrant metabolic activity [22,23]. Therefore, NAD^+^ metabolism is an emerging therapeutic target for the treatment and prevention of specific human disorders [9,10,11,24,25]. Moreover, hallmarks of cellular aging, including mitochondrial dysfunction, reactive oxygen species (ROS) generation, and genomic instability, are correlated with autophagy and NAD^+^ dysregulation [26]. Mitochondria are energy-generating hubs in cells and are subject to high rates of damage [27]. Proper turnover of damaged mitochondria, or mitophagy, is required to prevent impacting the biosynthesis and catabolism of acetyl-CoA, NAD^+^, polyamines, fatty acids, and amino acids [27]. Nutrient signaling pathways such as TOR (Target of Rapamycin) [28,29,30,31,32], Snf1 (yeast ortholog of mammalian AMP-activated Protein Kinase) [33,34,35], and PKA (Protein Kinase A) [36,37] have been shown to regulate autophagy, but these regulatory circuits are poorly understood.

The budding yeast *Saccharomyces cerevisiae* has been at the forefront of many key discoveries involving NAD^+^, autophagy, and related pathologies. Many autophagy- and NAD^+^ metabolism-related proteins are conserved from yeast to humans; this model system may serve as a jumping-off point for translation to more complex systems like mammals. NAD^+^ is increasingly recognized as a potential regulator of autophagy, but their mechanistic interconnection is not clearly defined. Most evidence stems from broader regulatory circuits that often converge on nutrient sensing pathways. It remains unclear whether low NAD^+^ conditions alone are sufficient to induce autophagy, whether this induction is dependent on sirtuins, and how NAD^+^ precursor supplementation may impact this process. In this review, we will discuss the interconnection of NAD^+^ and autophagy through overlapping signaling pathways, regulatory mechanisms, and the impact of NAD^+^ precursor supplementation on autophagy. While this review will primarily discuss *S. cerevisiae*, we will also touch on recent discoveries in mammalian systems, discrepancies between the two systems, and the connection of NAD^+^ and autophagy to human health and diseases [38].

## 2. NAD^+^ Metabolism

### 2.1. Overview of NAD^+^ Biosynthetic Pathways

**Figure 1:**
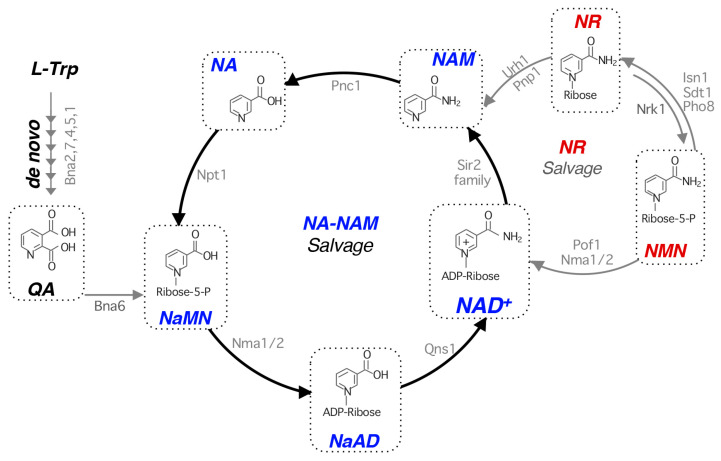
NAD^+^ biosynthesis pathways in yeast. Biosynthesis: the de novo pathway (**left panel**, simplified view) converts L-tryptophan (L-Trp) to quinolinic acid (QA) via Bna2, Bna7, Bna4, Bna5, and Bna1. QA is converted to nicotinic acid mononucleotide (NaMN) via Bna6. NA-NAM salvage (middle panel) also produces NaMN by conversion of nicotinamide (NAM) to nicotinic acid (NA) via Pnc1. Npt1 converts NA to NaMN, which is then converted to nicotinic acid adenine dinucleotide (NaAD) by the NMNATs, Nma1 and Nma2. NaAD is converted to NAD^+^ by Qns1. In NR salvage (**right panel**), nicotinamide riboside (NR) is converted to nicotinamide mononucleotide (NMN) by NR kinase Nrk1, which is then converted to NAD^+^ by Pof1, Nma1, and Nma2. Degradation and recycling: NAD^+^ is converted to NAM by the Sir2 family of sirtuins or to NMN by NADases. NMN is converted to NR nucleotidases/phosphatases Isn1, Sdt1, and Pho8. NR can be converted to NAM via nucleosidases Urh1 and Pnp1, which then enters NA-NAM salvage.

In yeast, NAD^+^ biosynthesis is facilitated by three pathways: de novo synthesis, NA (nicotinic acid)-NAM (nicotinamide) salvage, and NR (nicotinamide riboside) salvage (Figure 1). The de novo pathway converts tryptophan to ACMS (2-amino, 3-carboxymuconic semialdehyde), via the Bna (Biosynthesis of Nicotinic Acid) proteins, Bna2, Bna7, Bna4, Bna5, and Bna1, followed by spontaneous cyclization of ACMS to QA (quinolinic acid). Notably, Bna2, Bna4, and Bna1 require oxygen for their enzymatic activity, so cells grown under anaerobic conditions rely on NA-NAM salvage [39]. QA is then converted by Bna6 (QA phosphoribosyltransferase) to NaMN (nicotinic acid mononucleotide), converging on NA-NAM salvage. NaMN is then converted to NaAD (nicotinic acid adenine dinucleotide) via the dual specificity NaMN/NMN adenylyltransferases (NMNATs) Nma1 and Nma2 [40,41]. Finally, NaAD is amidated to NAD^+^ via the NAD^+^ synthetase Qns1 [42].

Under NAD^+^ replete conditions, the *BNA* genes are silenced by the NAD^+^-dependent histone deacetylase (HDAC) Hst1 [43,44]. When NAD^+^ is depleted, Hst1 activity is downregulated, and *BNA* genes are de-repressed. The mammalian analog of the de novo pathway is the kynurenine pathway that converts tryptophan to ACMS, from which QA is derived [45]. The importance of each pathway in NAD^+^ biosynthesis depends on growth conditions and may be tissue-specific, which is discussed at length elsewhere [5]. Although it is unclear whether the kynurenine pathway is similarly regulated by sirtuins and NAD^+^ abundance, there is evidence that sirtuins modulate the kynurenine pathway by regulating the expression of Indolamine 2,3-Dioxygenase 1 (IDO1) in bone marrow-derived dendritic cells [46].

In yeast, NA-NAM salvage is the preferred route when exogenous pyridines are available [47,48]. During exponential growth in standard yeast media, NAD^+^ is produced predominantly from the NA-NAM salvage pathway due to high concentrations of NA [47,48] and repression of *BNA* genes by Hst1 [43,44]. NA is directly taken up by the NA transporter Tna1 (Transporter of Nicotinic Acid), which is converted to NAMN by Npt1 (NA phosphoribosyltransferase) (Figure 1). Recycled NAM is converted to NA through Pnc1 (nicotinamidase). Tna1 can also import QA but has a higher affinity for NA, favoring NA import when it is abundant [47]. To date, a transporter of NAM has yet to be discovered in yeast. In humans, this pathway is known as the Preiss-Handler pathway and does not include the salvage of NAM [49,50]. Instead, NAM generated by NAD^+^-consuming enzymes, like sirtuins (NAD^+^-dependent HDACs) and PARPS (poly ADP-ribose polymerases), is converted to NMN (nicotinamide mononucleotide) via Nampt (NAM phosphoribosyltransferase) [51], which is then converted to NAD^+^ by NMNATs [51]. Although the Pnc1-like nicotinamidase is absent in mammalian cells, it is reported that gut microbiota help convert NAM to NA via the bacterial nicotinamidase, PncA [52]. Moreover, bacterial PncA also confers host cell resistance to NAMPT inhibitors by boosting NAD^+^ synthesis using administered NAM and NR in mammalian cells infected with *Mycoplasma hyorhinis* [52]. Interestingly, NAMPT, which converts NAM to NMN, has been implicated in autophagy, where the knockdown of NAMPT inhibits autophagic flux, and NAMPT overexpression activates in cardiomyocytes [53].

While NR salvage also contributes to the NAD^+^ pool, standard media do not contain NR due to its labile nature [54,55]. In NR salvage, NR is converted to NMN via the NR kinase, Nrk1 [56]. NMN is converted to NAD^+^ through the Nmnats Nma1, Nma2, and Pof1. Cellular NMN is constantly converted to NR through cytoplasmic and vacuolar nucleotidases Isn1, Sdt1, and Pho8 [55,57]. In addition, NR can be converted to NAM, entering NA-NAM salvage, through the enzymatic activities of the nucleosidases Urh1, Pnp1, and Meu1 [54]. When present in the growth media, NR is taken up by the NR transporter Nrt1 [58]. Yeast cells release small NAD^+^ precursors including QA, NA, NAM, and NR, which can be re-uptaken by their respective transporters. Moreover, the vacuole also plays a role in the storage and homeostasis of NAD^+^ precursors [59]. These precursors can be used to modulate NAD^+^ pools depending on nutrient and metabolic status.

### 2.2. Regulation of NAD^+^ Metabolism

NAD^+^ biosynthesis primarily occurs in the cytosol and the subcellular localization of NAD^+^, NADH, and precursor pools is highly regulated. NAD^+^ intermediates can also function as signaling molecules and high intracellular concentrations may inhibit certain cellular processes. Several proteins and pathways are known to regulate NAD^+^ metabolism. Sirtuins, like Sir2 and Hst1, consume NAD^+^ to generate NAM and ADP-ribose [60]. As such, NAM is a potent inhibitor of sirtuin activity [61]. For example, high NAD^+^ consumption, resulting in an increase in NAM, may cause unwanted transcription of *BNA* genes through Hst1 inhibition [43,44]. Compartmentalization of NAD^+^ pools also adds complexity to the regulation of NAD^+^ homeostasis. For example, mitochondrial NAD^+^ pools appear to be largely insensitive to cytosolic and nuclear NAD^+^ depletion [62]. NAD^+^ must be transported by specific mitochondrial transporters in both yeast and mammals [63,64,65]. Proper cytosolic and mitochondrial NAD^+^:NADH ratios are maintained through the NAD^+^/NADH redox shuttles including the malate-aspartate and glycerol-3-phosphate shuttles [66,67].

NAD^+^ metabolism is highly responsive to major cell signaling pathways in yeast, including the phosphate (*PHO*) signaling pathway, the amino acid and nitrogen-sensing TOR, and the cyclic AMP (cAMP)-dependent Protein Kinase A (PKA) [13,68]. Furthermore, purine metabolism modulates NAD^+^ biosynthesis via ATP concentration and the purine biosynthesis intermediates 5-amino-4-imidazole carboxamide ribonucleotide 5-phosphate (ZMP), and its precursor succinyl-ZMP (SZMP). Abundant adenine and ATP conditions support NAD^+^ biosynthesis via NA-NAM salvage. Under adenine deplete conditions, de novo NAD^+^ biosynthesis is induced by the Bas1-Pho2 complex in an (S)ZMP-dependent manner [69]. Recent studies have also connected the regulation of de novo NAD^+^ pathway to the copper-sensing transcription factor Mac1 [44] and to the interplay of the Bas1, Pho2, and Pho4 transcription factors that function in purine and phosphate metabolism. Interestingly, Mac1 appears to repress *BNA* gene expression. The putative chromatin remodeling activity of Mac1 was unexpected and its mechanisms remain to be further studied. The activity of the *PHO* activating transcription factor complex, Pho2-Pho4, is also promoted in an (S)ZMP-dependent manner. Low phosphate conditions induce a decrease in ATP and an increase in ZMP, stimulating both Pho2-Pho4 and Bas1-Pho2 complex formation [69,70]. The Bas1-Pho2 complex promotes genes in purine biosynthesis and de novo NAD^+^ synthesis, and it remains unclear whether these two complexes may compete for Pho2 under specific conditions. A recent paper demonstrated that the de novo pathway is regulated by the non-NAD^+^-dependent HDAC Rpd3, alongside Hst1 [71]. Rpd3 also appears to regulate NA-NAM and NR salvage, intersecting with the *PHO* pathway [71]. These studies suggest a complex interconnection and co-regulation of NAD^+^ metabolism and nutrient sensing. Detailed mechanisms remain to be further studied.

Recent studies in yeast have also linked the regulation of NAD^+^ metabolism to N-terminal acetylation, mediated by the NatB complex. NatB was identified as a novel NAD^+^ homeostasis factor, with NatB mutants showing altered levels of NAD^+^, NA, and NAM [18]. NatB regulates Nma1 and Nma2 (Nmnats), which are essential to NAD^+^ biosynthesis [72]. NatB-mediated N-terminal acetylation of Nma1 and Nma2 is required for maintaining proper protein levels, and the absence of NatB-mediated N-terminal acetylation leads to an approximately 50% reduction in Nmnats and NAD^+^ levels due to blunted protein maturation [72]. The results suggested that N-terminal acetylation on Nma1 and Nma2 is crucial for proper protein folding and/or stabilization during synthesis. These findings in yeast may help provide a mechanistic foundation for understanding how N-terminal acetylation regulates human NAD^+^ metabolism. While human NMNAT1 is predicted to be a potential NatB substrate [72,73], whether NatB-mediated regulation of NAD^+^ biosynthetic enzymes is conserved in humans remains unclear. Additionally, in mammals, NAD^+^ metabolism and sirtuins have been implicated in AMPK and mTOR signaling [74,75,76]. These pathways are key players in the regulation and induction of autophagy, to be discussed in Section 4.

## 3. Autophagy and NAD^+^ Metabolism

**Figure 2:**
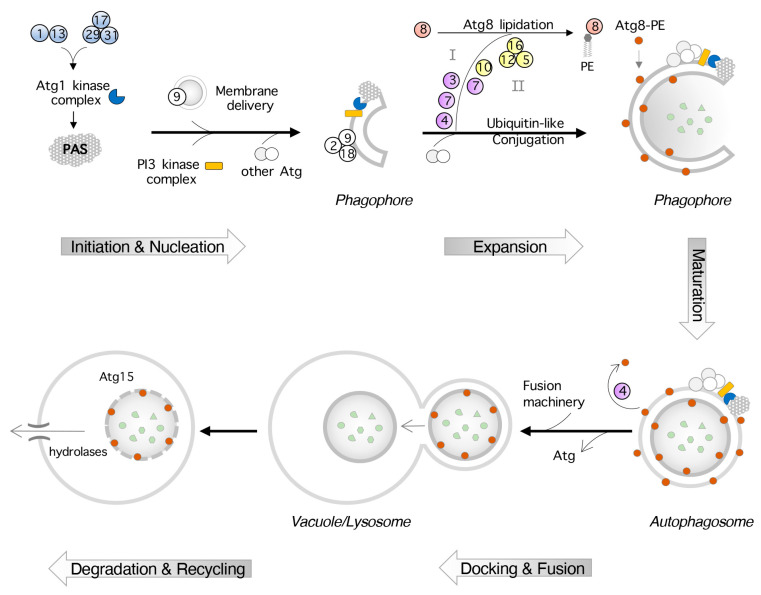
Overview of major steps in autophagy. Atg proteins are represented in circles with their corresponding number. Initiation and Nucleation: Autophagy is induced by the recruitment of the Atg1 kinase complex, consisting of the Atg1-Atg13 dimer and the Atg17-Atg29-Atg31 ternary subcomplex, to the phagophore assembly site (PAS). Atg9 delivers membrane to the phagophore. The phosphatidylinositol 3-(PI3) kinase complex deposits phosphatidylinositol-3-phosphate (PI3P) (not depicted) throughout the phagophore. Atg2, Atg9, and Atg18 recruit membrane to the expanding phagophore. Expansion: Atg8 and Atg12 are ubiquitin-like proteins used in the two (I and II) ubiquitin-like conjugation systems. The Atg12-Atg5-Atg16 complex conjugates Atg8 to PE. Atg8-PE (small red circles) is conjugated to PI3P on the membrane on both sides of the phagophore. Maturation: the phagophore fully sequesters the cargo, becoming the autophagosome. Atg4 cleaves external Atg8-PE. Docking and Fusion: Atg proteins disassociate, and fusion machinery facilitates docking of autophagosome to the vacuole (yeast) or lysosome (mammals). The inner membrane (autophagic body) is released to the vacuolar/lysosomal lumen. Degradation and Recycling: The autophagic body is degraded by Atg15 and cargo is recycled by hydrolases. Recycled cargo is exported to the cytosol.

Autophagy is ubiquitous to all eukaryotes, occurring constitutively at a basal rate, but can be upregulated in response to various stressors and functions in homeostatic regulation and organelle quality control. Autophagy is activated in response to starvation, nutrient depletion, and organelle damage to prevent further harm [77]. This process is tightly regulated at several levels to avoid excessive degradation, including epigenetic, transcriptional, post-transcriptional, translational, and post-translational. Autophagy-related proteins (Atgs) were first identified in yeast and are conserved in many eukaryotes, including humans [78]. In yeast and mammals, there are two types of autophagy: microautophagy and macroautophagy [79]. Both types can be further characterized as selective and non-selective, or bulk, autophagy [80]. Macroautophagy is the most studied and, generally, the proteins needed for macroautophagy are also required for microautophagy. Macroautophagy is hallmarked by the formation of double membrane vesicles, or autophagosomes, that sequester cytoplasmic cargo for transport to the vacuole (Figure 2) [79]. We will discuss macroautophagy, henceforth referred to as autophagy, in detail.

### 3.1. Overview of Bulk Autophagy

Bulk autophagy involves the non-specific sequestering of cytoplasmic contents and is often induced by starvation [81]. The formation of the autophagosome can be classified into four stages: initiation and nucleation, expansion and maturation, fusion to vacuole, and degradation and efflux of cargo [79]. Initiation begins proximal to the vacuole at the phagophore assembly site (PAS). Here, the Atg1 complex, consisting of Atg1, Atg13, and the ternary subcomplex (Atg17-Atg31-Atg29) are recruited to initiate nucleation [82,83]. The phosphatidylinositol 3-kinase (PtdIns3K) complex, consisting of Atg14, Vps34, Vps30, Vps15, and Vps38, is recruited to the PAS to deposit phosphatidylinositol-3-phosphate (PI3P) throughout the phagophore [84]. Phagophore expansion is mediated by two ubiquitin-like (Ub1) conjugation systems that conjugate Atg12 and Atg8. Atg12 is activated by Atg7 (E1-like) to conjugate Atg12 to Atg5 in an Atg10 (E2-like)-dependent manner [85,86]. The Atg12-Atg5 complex is associated with Atg16 to facilitate membrane recruitment to the phagophore [87]. Atg4 cleaves the C-terminal arginine residue on Atg8, leaving an exposed glycine residue [88]. The Atg12-Atg5-Atg16 complex (E3-like) conjugates Atg8 to the lipid phosphatidylethanolamine (PE) [89]. Atg8-PE is conjugated to the PI3P motifs on both sides of the phagophore (Figure 2).

Once the cargo has been fully engulfed, the phagophore, now autophagosome, may undergo maturation and the outer Atg8-PE is deconjugated by Atg4 [90]. While the role of Atg9 is yet to be elucidated, the transmembrane protein undergoes cycling between the PAS and peripheral sites and may facilitate membrane recruitment during expansion, alongside Atg2 and Atg18 [91,92]. The outer membrane of the matured autophagosome fuses with the vacuole, releasing the inner membrane to the lumen. The resulting autophagic body is lysed by Atg15, a vacuolar lipase [93]. Vacuolar hydrolases degrade the released contents, and the products are exported to the cytosol by vacuolar membrane permeases like the amino acid transporter Atg22 [94].

Autophagy has also been extensively studied in mammalian systems where there are a few key differences compared to yeast. Namely, the autophagosome fuses with the lysosome rather than the vacuole [95]. Further divergence involves autophagy regulation by PARPs in mammals, which are absent in yeast, and has been discussed elsewhere [6]. Another major NAD^+^-consuming enzyme, the ADP-ribosyl cyclase/cyclic ADP-ribose hydrolase CD38, is also absent in yeast. Both PARP1 and CD38 have been implicated in age-associated NAD^+^ decline [5]. An increase in the expression and activity of CD38 results in a decline in NAD^+^, leading to mitochondrial dysfunction in aged mice in a sirtuin (SIRT3)-dependent manner. While it is unclear if CD38 directly regulates autophagy, it may be involved in lysosomal integrity (discussed further in [6]). In addition to CD38, other NAD^+^-consuming enzymes have been identified in mammals. Among them, SARM1 is an NAD(P) glycohydrolase that cleaves NAD^+^ upon neuronal injury. Its function is critical for response to nervous system injury, resulting in a rapid decrease in axonal NAD^+^ [7]. Excessive SARM1 activity may lead to NAD^+^ depletion, causing axonal degeneration [8]. Interestingly, ULK1 directly regulates SARM1 during axonal injury, contributing to SARM1 accumulation [7]. SARM1 has been discussed further elsewhere [8].

### 3.2. Overview of Selective Autophagy

Selective autophagy is characterized by targeted sequestering of specific cellular components including organelles and protein aggregates [96]. For example, mitophagy is a type of selective autophagy that degrades mitochondria. Mitochondria are susceptible to oxidative damage and mitophagy is a key mediator of oxidative stress response [97]. In budding yeast, this process generally follows the same steps as non-selective autophagy with a few differences. Rather than the bulk engulfment of cytoplasmic contents, the autophagosome recognizes mitochondria through interactions with Atg11 and Atg32. Atg32, a mitophagy receptor in budding yeast that localizes on the outer mitochondrial membrane, interacts with Atg11 upon mitophagy induction [98,99]. This interaction is stabilized by the phosphorylation of two serine residues on Atg32, and Atg11 tethers the mitochondria to the PAS via Atg1 and Atg9 [100]. Most selective autophagy receptors, including Atg32, contain the Atg8 family interacting motif (AIM) that is used to interact with Atg8 and facilitate sequestering of mitochondria [101]. In mammalian systems, this process is significantly more complex. Most systems contain several pathways that can compensate for the loss of one another, including the phosphatase and tensin homolog (PTEN)-induced kinase 1 (PINK1) and the cytosolic E3 ubiquitin ligase Parkin, which have both been implicated in Parkinson’s disease [102]. These mechanisms have been discussed in detail elsewhere [27].

The cytoplasm to vacuolar targeting (Cvt) pathway, another form of selective autophagy, is only present in yeast. This pathway is the only biosynthetic pathway that uses autophagic machinery and delivers the hydrolases alpha-mannosidase (Ams1) and aminopeptidase I (Ape1) to the vacuole [103]. Ape1 is an inactive proenzyme (prApe1) until activated by the removal of its propeptide in the vacuole [104]. This process involves core autophagy machinery, and largely follows that of nonspecific autophagy, but is predicated by the formation of the Cvt complex. The Cvt complex consists of a dodecamer of prApe1, oligomers of Ams1, and Atg19 which binds to Atg11 to facilitate transport of the complex to the PAS on actin cables. Atg11 and Atg19 are proteins specific to selective autophagy and are not part of the core autophagic machinery. Atg11 appears to function as an adapter protein, binding the cargo receptor, in this case Atg19, to Atg8 [104]. Interestingly, Atg21, a homolog of Atg18, functions specifically in the Cvt pathway [105]. Both Atg18 and Atg21 appear to be required for efficient autophagosome formation and shield Atg8-PE from premature cleavage by Atg4 [105]. The Cvt pathway has been discussed at length elsewhere [104,106]. There are many other forms of selective autophagy that degrade organelles including peroxisomes (pexophagy), nuclei (nucleophagy), and ribosomes (ribophagy) that have been discussed in detail elsewhere [107].

### 3.3. Regulation of Autophagy by Acetylation

#### 3.3.1. Overview of Protein Acetylation

Acetylation is a post-translational modification that affects protein function, influencing metabolism and gene expression. It is also an important regulatory mechanism for autophagy, dictating initiation and autophagosome formation [108,109,110]. Two types of acetylation, lysine acetylation and N-terminal acetylation, both occur in many cellular processes [12,111] and have been implicated in the regulation of autophagy and NAD^+^ metabolism (discussed in Section 2.2). In yeast, lysine acetylation-mediated regulation of autophagy gene expression largely occurs though remodeling the chromatin at *ATG* gene promoters by histone acetyltransferases (HATs) and HDACs, like sirtuins. Moreover, the level of acetylation is intrinsically connected to acetyl-CoA abundance. For example, in yeast, *ATG7* expression is repressed when nucleocytosolic acetyl-CoA levels are high, leading to increased histone acetylation [112]. In mammalian systems, the sirtuin SIRT1 deacetylates lysine residues on multiple ATG proteins, including ATG5, ATG7, and LC3 (mammalian ATG8), which is essential for their proper function and autophagy induction [113,114]. Additional details are discussed in Section 3.3.2 and Section 4.4.

Moreover, N-terminal acetylation by NatB is particularly crucial for autophagy machinery assembly and NAD^+^ biosynthetic enzyme stability [18,72,115,116,117,118]. N-terminal acetylation occurs in two steps. First, methionine aminopeptidases (MetAPs) determine whether to remove the initiating methionine based on the size of the second amino acid. Then, N-acetyltransferase (NAT) complexes catalyze the covalent attachment of an acetyl group (CH_3_CO) from acetyl-CoA to the free alpha-amino group (NH_3_^+^) at the N-terminal end of the substrate, forming a stable amide bond and neutralizing the positive charge [108]. Each NAT enzyme possesses a distinct subunit composition and substrate specificity. Major NAT complexes include NatA, NatB, and NatC, and consist of one catalytic subunit and one auxiliary subunit, serving as a ribosomal anchor [119]. The NatB complex directly links N-terminal acetylation to autophagy regulation and NAD^+^ homeostasis, making it crucial to understanding the metabolic control of cellular homeostasis [108,109,110]. Act1, involved in actin filament formation and required for vesicle transport, and Vps1, important for autophagosome-vacuole fusion, are both targets of NatB-mediated N-terminal acetylation [115]. The involvement of Act1 and Vps1 in autophagy and their N-terminal acetylation by the NatB complex was further verified by studying constitutively acetylated Act1 and Vps1 mutants. However, it was also shown that constitutively acetylated Vps1 and Act1 could not restore autophagy in *nat3Δ* cells defective in NatB [115]. The authors suggest that there are undiscovered NatB substrates that function in autophagosome fusion, in addition to Vps1. A few earlier studies show that the NatB complex also acetylates Tpm1, a yeast tropomyosin involved in actin stabilization [116,117,118].

**Figure 3:**
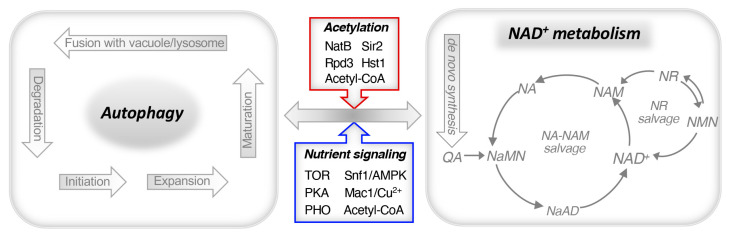
Factors regulating NAD^+^ metabolism and autophagy. Nutrient signaling pathways (**lower box**) that affect both NAD^+^ metabolism and autophagy: TOR, PKA, PHO, Snf1/AMPK, Mac1/Cu^2+^, and acetyl-CoA. Acetylation by proteins (**upper box**) that affect both NAD^+^ metabolism and autophagy: NatB, Rpd3, Sir2, and Hst1 using acetyl-CoA. Arrows between autophagy (**left**) and NAD^+^ metabolism (**right**) indicate interplay between pathways. Arrows between Acetylation and Nutrient Signaling indicate that regulation of nutrient signaling pathways by acetylation and regulation of acetylation factors by nutrient signaling pathways.

Autophagy induction is regulated by several pathways that have also been implicated in the regulation of NAD^+^ metabolism, suggesting some degree of crosstalk between these two pathways (Figure 3). Table 1 provides a summary of yeast and mammalian autophagy proteins regulated by sirtuins and nutrient-sensing pathways that also play a role in NAD^+^ metabolism. In subsequent sections, we will discuss the role of sirtuins, *PHO* signaling, TOR, PKA, and Snf1/AMPK in NAD^+^ metabolism and autophagy.

#### 3.3.2. Regulation of Autophagy by Sirtuins

Sirtuins (Sir2) are a family of NAD^+^-dependent deacetylases that are conserved from bacteria to humans, with a core domain essential for their enzymatic function [120]. Yeast has five sirtuins, Sir2 and Hst1-4, and humans have seven, SIRT1-7 [120]. Sirtuins are primary sources of NAD^+^ consumption in yeast and mammals, cleaving NAD^+^ to ADP-ribose and NAM [121]. As an NAD^+^-dependent deacetylase, sirtuin activity is tightly regulated by NAD^+^ availability. The age-associated decrease in NAD^+^ availability is often accompanied by a decrease in sirtuin activity [122]. PARPs, which are absent in yeast, indiscriminately cleave NAD^+^ in response to DNA damage, contributing to age-related NAD^+^ depletion [123]. Uncontrolled NAD^+^ cleavage by PARPs is proapoptotic and has been discussed in detail elsewhere [6]. It is important to note that PARP hyperactivation, and subsequent NAD^+^ consumption, can result in loss of sirtuin activity and abnormal autophagy [124]. Sirtuin activity is generally correlated with an increase in lifespan and healthspan in yeast, fruit flies, nematodes, and mice and is correlated with an increased stress response [122]. A precise balance between cleavage and synthesis is crucial for the maintenance of NAD^+^ pools, metabolism, and the acetylation status of sirtuin targets.

In yeast, Sir2 is required for proper silencing of ribosomal DNA repeats, telomeres, and the silent mating type loci [125] as well as segregation of damaged proteins during budding [126]. Hst2 appears to be the most abundant sirtuin in yeast, performing the bulk of the NAD^+^-dependent deacetylase activity [3]. In mammalian systems, sirtuins function through the deacetylation of both histone and non-histone proteins, including the tumor suppressor p53, the forkhead box protein O1 (FoxO1), and histone 3 lysine residue 9 (H3K9) that is responsible for NF-kB expression [127,128]. Notably, sirtuins play a key role in the regulation of transcription factors and proteins involved in autophagy and deacetylation of these targets is regarded as the central inducer of autophagy [129]. In yeast, this connection was mainly shown through deletion and overexpression of Sir2, which positively modulates *ATG* gene expression. The direct impact of Sir2 on *ATG* gene expression has also been shown in different model systems. For example, overexpression of Sir2 counterparts in nematodes upregulates autophagy [130]. Recent studies further supported Sir2’s role in maintaining *ATG* gene expression during aging and demonstrated that Sir2 deacetylates Atg8a during starvation to activate autophagy in *Drosophila* [131]. The hypoacetylation of histones during aging correlates with the enhanced expression of ATGs, promoting autophagy induction [132]. Furthermore, a rise in the expression of SIRT1 is sufficient to increase basal rates of autophagy via the deacetylation of ATG5 and ATG7 [113]. SIRT1 also functions in the deacetylation of LC3, inducing its translocation from the nucleus to the cytosol to participate in autophagosome formation at the PAS [113,133]. In a similar vein, Rpd3, a non-NAD^+^-dependent HDAC, has been implicated in the transcriptional control of autophagy-related proteins including Atg8 and Atg9 in yeast [29,134,135]. Despite not requiring NAD^+^ for its activity, Rpd3 has been shown to function alongside Hst1 in the regulation of the de novo pathway, providing another connection between autophagy and NAD^+^ metabolism [71].

Sirtuins also play an important role in senescence, or stable cell cycle arrest, that is induced by telomere shortening and stress [136]. Aged tissues are characterized by an accumulation of senescent cells in mammals and the clearance of these cells may delay age-related pathologies [137]. Autophagy may be pro- or anti-senescence, depending on the context. A previous review highlighted the importance of context in studies examining the connection of autophagy to senescence [138]. In particular, the effects of autophagy on senescence may depend on the method and duration of induction/suppression. Basal autophagy appears to suppress irradiation-induced senescence, while the inhibition of autophagy may suppress oncogene-induced senescence. Prolonged inhibition, however, may induce senescence [138]. While basal autophagy may be protective, the effect of modulating autophagy likely depends on the method, duration, and model used.

Notably, in yeast, the Sir2 protein level is known to decline upon replicative aging [139]. It has been shown that Sir2 is required for the transcriptional upregulation of both *ATG8* and *ATG32* during chronological aging with Sir2 deletion, resulting in the strongly reduced expression of these genes, particularly *ATG32* [140]. The effect of Sir2 on Atg8 and Atg32 protein concentration was not monitored. Additionally, the effect of replicative senescence on Sir2, Atg8, and Atg32 was not examined. The SIRT1 protein is also known to be reduced upon senescence [136] and the overexpression of mammalian SIRT1 and yeast Sir2 has been shown to delay replicative senescence in aging cells [141,142].

Nuclear SIRT1 is subjected to clearance by autophagy in an LC3-dependent manner in senescent cells [114]. Deacetylation of LC3 enhances the LC3-SIRT1 interaction during senescence, promoting the degradation of SIRT1. *SIRT1* mRNA appears to increase in oncogene-induced senescence but decreases in replicative senescence. However, a decrease in SIRT1 protein by LC3 is observed in both models. This association has yet to be observed in yeast. There is no change in *SIRT1* mRNA in young and aged mice, but a decrease in SIRT1 protein is observed in aged mice which is restored by the suppression of autophagy. The discrepancy between *SIRT1* mRNA and protein suggests a role for post-transcriptional modifications. It remains to be determined if other sirtuins are also degraded by autophagy upon senescence or if this mechanism is unique to SIRT1. Additionally, autophagy itself was not measured in this study, so it is unknown how degradation of SIRT1 in senescence impacts autophagy. It is possible that a decrease in SIRT1 may result in a loss of autophagy over time.

Further questions remain regarding the role of Sir2 in yeast and SIRT1 in mammals. Sir2 and SIRT1 appear to be involved in autophagy gene transcription and SIRT1 is also involved in autophagy protein modification, however SIRT1 is degraded by autophagy. It is possible that the role of mammalian SIRT1 is more complex than that of its yeast counterpart. Whether these differences reflect evolutionary divergence between the two models or whether this is simply underexplored remains unclear, and direct comparison is further complicated by the differences in model systems, cell types, and methods employed to modulate autophagy and senescence.

**Table 1:** Yeast and mammalian autophagy-related proteins regulated by sirtuins and nutrient sensing pathways that also play a role in NAD^+^ metabolism.

Step	Complex	Target	Regulated by	Type	System	Reference
Initiation and Nucleation	*Atg1* *Kinase* *Complex*	Atg1/ULK1	Pho23-Rpd3	Gene expression	Yeast	[135]
			TORC1 (via * TFs)	Gene expression	Yeast	[143]
			mTORC1	Protein modification	Mammalian	[144]
			AMPK & mTORC1	Protein modification	Mammalian	[145,146,147]
			PKA	Protein modification	Yeast	[36,148]
		Atg13	TORC1	Protein modification	Yeast	[30,135]
			TORC1 (via TFs)	Gene expression	Yeast	[143]
			Snf1	Protein modification	Yeast	[33]
		Atg29	TORC1 (via TFs)	Gene expression	Yeast	[143]
Expansion and Maturation	*Ubiquitin-like* *conjugation*	Atg7	SIRT1	Both	Mammalian	[113]
			Pho23-Rpd3	Gene expression	Yeast	[135]
			TORC1 (via TFs)	Gene expression	Yeast	[143]
		Atg5	SIRT1	Both	Mammalian	[113]
		Atg8/LC3	SIRT1	Both	Mammalian	[113]
			SIRT1	Protein modification	Mammalian	[113,133]
			Ume6-Sin3-Rpd3	Gene expression	Yeast	[29,134,135]
			Pho23-Rpd3	Gene expression	Yeast	[135]
			Rpd3L	Gene expression	Yeast	[29,134,135]
			TORC1 (via TFs)	Gene expression	Yeast	[143]
	*PI3K*	Atg14	Pho23-Rpd3	Gene expression	Yeast	[135]
			TORC1 (via TFs)	Gene expression	Yeast	[143]
	*Vesicular*	Atg9	Pho23-Rpd3	Gene expression	Yeast	[135]
			Rpd3L	Gene expression	Yeast	[29,134,135]
			TORC1 (via TFs)	Gene expression	Yeast	[143]
			AMPK & mTORC1	Protein modification	Mammalian	[147]
Efficient Autophagosome formation	*N/A*	Atg41	TORC1 (via TFs)	Gene expression	Yeast	[143]
			TORC1 (via TFs)	Gene expression	Yeast	[31,143]
Selective Autophagy	*Mitochondrial*	Atg32	TOR and Rpd3	Gene expression	Yeast	[80]
			TORC1	Protein modification	Yeast	[149]
			TORC1 (via TFs)	Gene expression	Yeast	[143]
Trafficking and Fusion	*Actin cables, Vacuolar*	Act1, Vps1	NatB	Protein modification	Yeast	[115,116,117,118]

* TFs: transcription factors

## 4. Nutrient Signaling Pathways That Regulate Both NAD^+^ Metabolism and Autophagy

Autophagy is primarily regulated in response to varying nutrient conditions, so it is no surprise that several autophagy-related proteins are under the control of major nutrient-sensing pathways. NAD^+^ metabolism is associated with several of these pathways, including *PHO* and TOR in yeast and mTOR and AMPK in mammals. Caloric restriction has been connected to the regulation of these pathways in various systems. For example, moderate caloric restriction, induced by limiting glucose content in media from 2% to 0.5%, has been shown to extend replicative lifespan in yeast in a Sir2 and NAD^+^-dependent manner [150]. Moderate caloric restriction shunts central carbon metabolism from fermentation to respiration in yeast [151]. Lifespan extension has also been observed in mammals, resulting in various metabolic and cardiovascular benefits, but the exact mechanism has not been identified and is discussed at length elsewhere [27]. While caloric restriction can extend longevity, it may be inhibited by the downregulation of Atgs [15]. In cell lines defective in autophagy, caloric restriction fails to elicit any benefits and may result in harmful side effects [152]. Importantly, caloric restriction requires that most nutritional demands are met [153].

### 4.1. Nitrogen and Amino Acid Sensing

Amino acids are present in distinct pools that are tightly regulated. In particular, the vacuolar pool is rich in basic and neutral amino acids that may serve as a source of nitrogen and precursors for carbon metabolism and nucleotide biosynthesis [14]. In nitrogen and amino acid starvation conditions, autophagy deficient cells have less intracellular amino acids, causing a decrease in protein synthesis [154]. Similarly, *ATG7*-deficient tumor-derived cell lines require nucleoside supplementation to suppress starvation-induced cell death, indicating that autophagy plays a key role in nucleotide homeostasis during starvation [155]. This was also observed in yeast where *ATG7*-deficient cells result in an imbalance in nucleotide turnover and an increase in mitochondrial ROS generation [156]. Amino acids also play a role as signaling molecules, communicating information to TOR and Snf1 (yeast AMPK) [14]. Like in mammals, rapamycin induces autophagy in yeast via TOR [28].

Yeast TOR complex 1 (TORC1) is associated with the vacuole and is considered the master regulator of nutrient sensing [157]. Mammalian and yeast TORC1 are responsible for stimulating protein synthesis and other forms of anabolism while suppressing catabolic functions, like autophagy, in nutrient-rich conditions [158]. In nutrient replete conditions, TORC1 is activated and localizes across the vacuolar membrane. In response to starvation conditions, TORC1 is inactive and exists as a singular punctate structure [159]. To date, it is unclear if this localization contributes to autophagy induction, but it is hypothesized that its dispersal along the vacuolar membrane may inhibit the recruitment of Atg13, a component of the Atg1 kinase complex, to the PAS [32]. Conversely, in mammals, inactive mTORC1 localizes to the lysosome while active mTORC1, under conditions of amino acid sufficiency, facilitates its release from lysosomes. The activity states of mTORC1 are more nuanced and may not be strictly defined as active and inactive, but rather more or less active towards specific targets, under specific conditions [160]. When nutrients are available, yeast TORC1 represses autophagy through the phosphorylation of Atg13, preventing the formation of the Atg1 complex [30]. When nutrients are limited, Atg13 is rapidly dephosphorylated by the PP2C phosphatases Ptc2 and Ptc3 which function downstream of TORC1 [161]. TORC1 also regulates the expression of the transcription factors Gcn4, Gln3, and Gat1 which regulate several *ATG* genes [162,163]. Gln3 and Gat1 are GATA type transcription factors that translocate to the nucleus to induce expression of *ATG7*, *ATG8*, *ATG9*, *ATG29*, and *ATG32* in response to nitrogen starvation [143]. Under amino acid starvation, Gcn4 regulates *ATG1*, *ATG13*, and *ATG14* as well as *ATG41*, which is required for efficient autophagosome formation [31,143].

In mammals, mTORC1 is also considered the master regulator of autophagy in response to nutrient stress. mTORC1 senses free amino acids in the cytosol and lysosomal lumen and controls the phosphorylation status of ULK1 (mammalian Atg1) and ATG13, inhibiting autophagosome formation [145,164]. Compared to TORC1, little is known about TORC2. Tor2 functions mainly on the plasma membrane and on endosomes [165]. TORC2 has been implicated in promoting autophagy in response to amino acid limitation in a manner independent of TORC1 [166]. TORC2 acts on the kinase Ypk1 to inhibit calcineurin, the Ca^2+^ and Cmd1/calmodulin-dependent phosphatase, which activates Gcn2 to promote autophagy [166]. This may indicate that calcium homeostasis plays a role in autophagy, but the degree in which remains to be determined. Yeast TORC1 appears to exert regulatory control on NAD^+^ metabolism via Pnc1 in the NA-NAM salvage pathway through the transcription factors Msn2 and Msn4 [167]. It is unclear how mTORC1 may play a role in the regulation of NAD^+^ metabolism.

### 4.2. Glucose Sensing

While both nitrogen and carbon limitation induce autophagy, nitrogen starvation typically results in more pronounced autophagy induction compared to glucose limitation [14,168]. However, how cells respond to nitrogen starvation and glucose starvation is modulated by additional factors, including specific growth conditions, genetic backgrounds, and experimental procedures. For example, autophagy facilitates adaptation from fermentation to respiration through recycling of serine for one-carbon metabolism in *S. cerevisiae* [169]. Additional nutrient stress, including metal, phosphate, and sulfur limitation, can also trigger autophagy and their respective regulatory pathways [170]. In some instances, glucose starvation conditions can inhibit nitrogen starvation-induced autophagy through the induction of vacuolar hydrolysis pathways [171].

A nutrient sensor critical for cellular adaptation to energy limiting conditions is AMPK, an evolutionarily conserved serine/threonine protein kinase that controls ATP turnover and metabolism [172,173]. Upon glucose starvation, ATP is decreased, promoting AMPK activity which promotes autophagy by phosphorylating associated proteins [174]. Unlike mammalian AMPK, yeast AMPK, Snf1, is not allosterically activated by AMP and instead correlates with a high AMP:ATP ratio [175]. In glucose-rich conditions, Snf1 is largely cytosolic, while in low glucose conditions, Snf1 localizes to the nucleus and mitochondria [176]. Mitochondria are regulatory hubs of glucose starvation-induced autophagy, similar to how the vacuole functions in nitrogen and amino acid-induced autophagy [177]. In the mitochondria, Snf1 phosphorylates Mec1, a genome integrity checkpoint protein, which recruits Atg1 and Atg13 to the mitochondria to facilitate the interaction between the mitochondria and the autophagosome [33]. It is hypothesized that the Snf1 localizes to mitochondria because they are the primary site of ATP generation and may be a source of materials required to form the autophagosome [33].

AMPK can directly phosphorylate several autophagy proteins, including ULK1, BECLIN1 (mammalian Atg6), and PIK3C3/VPS34 [145,146]. Interestingly, SIRT1 is also under the regulation of AMPK and is activated by increasing NAD^+^ concentrations in response to starvation. The deacetylation of ATG5 and LC3 by SIRT1 may function to further upregulate autophagy [113]. Furthermore, AMPK can be inhibited by mTORC1, contributing to even more interconnection between these pathways [178]. This degree of interconnection has yet to be observed in yeast. Like AMPK and Snf1, the cAMP-dependent serine-threonine kinase, PKA is activated by glucose to modulate cell growth, metabolism, and stress response [179]. Notably, yeast PKA cannot inhibit autophagy alone. Rather, Ras-PKA negatively regulates autophagy via the phosphorylation of Atg1 and Atg13, preventing their localization to the PAS [36]. This also requires the inhibition of the TORC1 substrate Sch9 (yeast Akt), indicating some degree of synergy between TORC1 and PKA on autophagy induction [180]. PKA, Sch9, and TOR have been shown to affect the NA-NAM salvage enzyme, Pnc1, through the transcription factors Msn2 and Msn 4 [68]. There is additional evidence that the *PHO* signaling pathway may modulate other signaling pathways like Ras-PKA, TOR, and Sch9 which has been discussed in [181].

### 4.3. Phosphate Sensing

Many proteins involved in autophagy are phosphorylated, which can have an activating or suppressing effect. Phosphorylation is the most frequent modification on autophagy proteins and serves as a fast on and off switch in response to stress. Atg1 is highly regulated by phosphorylation by PKA or through autophosphorylation [148]. Likewise, ULK1 can also be autophosphorylated or phosphorylated by AMPK and mTORC1 [147]. This suggests autophagy may be intertwined with phosphate signaling, also known as the *PHO* pathway in yeast. Once phosphate pools are depleted, autophagy is induced, however the extent in which is lower than that of nitrogen starvation [182]. Deletion of Pho81 can increase the demand for autophagy during phosphate starvation through the inhibition of the Pho80-Pho85 kinase complex that functions to sense internal and external phosphate [183]. Under phosphate replete conditions, Pho80-Pho85 phosphorylates Pho4, which induces its export out of the nucleus [184]. Under phosphate limitation, Pho81 inhibits the Pho80-Pho85 complex, resulting in accumulation of unphosphorylated Pho4 in the nucleus. This permits Pho4 to activate the expression of *PHO* genes, including the phosphatases Pho5 and Pho8, the high affinity phosphate transporters Pho84 and Pho89, and factors involved in the mobilization of vacuolar phosphate storage [184]. Furthermore, we have previously shown that the NAD^+^ precursor NMN can be dephosphorylated by Pho5 and can serve as a source of phosphate [55]. Phosphate signaling may not always be governed by *PHO*, but also by TORC1 where low phosphate conditions cause Atg13 to be dephosphorylated, initiating autophagy dependent on Atg11 [182]. How this is sensed and fed to TORC1 and autophagy proteins remains unknown.

In yeast, *PHO* signaling plays a role in each branch of NAD^+^ biosynthesis, to varying degrees. Cells grown under low phosphate conditions and cells lacking Pho84, a genetic mimic of low phosphate, display an increase in internal and released NR mediated by *PHO* signaling [59]. Furthermore, it was shown that *PHO* signaling responds to the depletion of intracellular NaMN where NaMN accumulation delays *PHO* activation and depletion enhances [59]. Low NaMN is reflective of low NAD^+^ levels and may signal the de-repression of *PHO* genes in an Hst1-dependent manner [59]. Pnc1 deletion mutants are characterized by increased intracellular and released NAM, but do not exhibit a decline in NAD^+^ [59]. These mutants exhibit *PHO* gene de-repression via Hst1 inhibition, likely mediated by an accumulation of NAM rather than a reduction in NAD^+^ [70]. Beyond linking NAD^+^ metabolism and phosphate sensing, the level of NaMN may function as a molecular signal to switch between NA-NAM and NR salvage, as a decrease in NaMN content results in the de-repression of NR salvage [59]. This may be a means to limit NR salvage, which requires utilization of ATP for its phosphate moiety, in favor of NA-NAM salvage. Interestingly, under phosphate replete conditions, Pho84 is degraded in a PKA-dependent manner indicating some degree of intersection between phosphate and glucose sensing pathways [185,186].

Rpd3, a non-NAD^+^-dependent HDAC (a human HDAC1 homolog), has also been implicated in the regulation of NAD^+^ biosynthesis. The deletion of Rpd3 results in a decrease in de novo pathway metabolites, opposite the deletion of the NAD^+^-dependent HDAC, Hst1 [71]. Hst1 and Rpd3 were found to function in chromatin restructuring in the promoter region of *BNA2*, where Hst1 represses this region and Rpd3 de-represses [71]. Mutants lacking Rpd3 were also observed to have an impact on the regulation of NA-NAM and NR salvage pathways. This mutant exhibits a decrease in *NPT1* and *PNC1* expression, resulting in a decrease in NAD^+^ and an increase in NA and NAM, respectively [71]. In addition, Rpd3 mutants display a decrease in gene expression of the NA transporter *TNA1*, the NR transporter *NRT1*, and *URH1*, an enzyme that converts NR to NAM. Conversely, mutants lacking Hst1 exhibit an increase in *TNA1* and *NRT1* expression and a decrease in *PNC1* expression. Increased *NRT1* expression in Hst1 mutants causes an accumulation of intracellular NR, while Rpd3 mutants show an increase in extracellular NR [187]. Cells lacking both Rpd3 and Hst1 show significant increases in intracellular NR, larger than that of both the single mutants, indicating a synergistic effect of these HDACs on NR salvage. Hst1 deletion mutants are also characterized by an increase in NA-NAM content, likely caused by an increase in transport of NA, via Tna1, and the repression of the de novo pathway. Under NA replete conditions, Hst1 represses the *BNA* genes in favor of NA-NAM salvage. Notably, the low NAD^+^ content in *rpd3∆* mutants may have an inhibitory effect on Hst1 but, as Hst1 functions downstream of Rpd3, additional factors are likely involved [71].

In a subsequent study, Hst1, Rpd3, and the transcription factor Pho2 were linked to regulation of *BNA* and *PHO* genes as well as NR and NA-NAM salvage [70]. The Pho2-Pho4 complex regulates *PHO* genes and the Bas1-Pho2 complex activates *BNA* genes under low adenine conditions, leading to postulate that Pho2 may be a limiting factor and the sharing of Pho2 may serve to coordinate these pathways. While Bas1-Pho2 is not a major regulator of the *BNA* pathway under standard conditions, it is required for full induction of this pathway under adenine deplete conditions [70], including in strains lacking *ADE16* and *ADE17*, a genetic mimic of adenine depletion [69]. Cells under phosphate starvation are characterized by a decrease in cellular NAD^+^ content. Notably, several steps in NA-NAM salvage require ATP making it more costly than de novo synthesis under phosphate starvation [69]. Cells lacking both Rpd3 and Hst1 exhibited significantly increased gene expression and enzymatic activity of the phosphatase Pho5 [70]. Conversely, *rpd3∆*, *hst1∆*, and *hst1∆rpd3∆* mutants exhibit similar activation effects on the phosphatase Pho8 [70]. As such, phosphate-limiting conditions may redirect NAD^+^ biosynthesis to the de novo pathway by promoting *BNA* expression via Bas1-Pho2 and the repression of Hst1 activity caused by low NAD^+^ abundance. Bas1-Pho2 and Pho2-Pho4 may aid in this by decreasing the expression of *PNC1*. Rpd3 positively regulates the *BNA* genes and *PNC1* while negatively regulating Pho5 and Pho8. This may serve to keep the *PHO*, de novo, and salvage pathways in check under NAD^+^ and phosphate limiting conditions [70].

In nutrient-rich conditions, Pho23, a component of the Rpd3L complex, represses the expression of several *ATG*s including *ATG1*, *ATG7*, *ATG8*, *ATG9*, and *ATG14* in an Rpd3-dependent manner [135]. Under nitrogen starvation, most *ATGs* are de-repressed via Pho23 except for *ATG9*, which continues to be repressed [135]. Cells lacking Pho23 tend to have more autophagosomes and higher autophagic activity in response to starvation [135]. As such, Atg9 appears to control the frequency of autophagosome formation. However, the Pho23-Rpd3 complex has also been shown to regulate the expression of *STB5*, a transcription factor that negatively modulates autophagy, with *STB5* expression increased in both *rpd3∆* and *pho23∆* mutants [188,189]. These results indicate Rpd3 has a complex role in autophagy regulation which remains to be further determined. The intimate link between *PHO* signaling, Rpd3L, sirtuins, and NAD^+^ metabolism has been further discussed elsewhere [59,70,181].

### 4.4. Acetyl-CoA

It has been presented that decreased levels of acetyl-CoA are associated with the activation of autophagy and mitophagy [27,77,112]. Acetyl-CoA sequestered in different subcellular compartments can differentially influence cellular processes. The TCA cycle is one example where acetyl-CoA oxidation is dependent on NAD^+^ availability, which serves as a coenzyme [9]. The regulation of acetyl-CoA is also connected to NAD^+^-dependent sirtuin activity, particularly Sir2/SIRT1, which controls acetyl-CoA synthesis through deacetylation and activation of acetyl-CoA synthetases [190]. Since acetyl-CoA serves as the primary substrate for acetylation, the level of acetyl-CoA is expected to impact the regulation of protein acetylation mediated by HAT/HDAC and N-terminal acetylases; however, the mechanisms remain to be further studied [191]. A mechanistic link between acetyl-CoA metabolism and autophagy was demonstrated in an earlier study showing that the depletion of the nucleocytosolic pool of acetyl-CoA promotes autophagy and lifespan in yeast and *Drosophila* [112]. These results indicate that nucleocytosolic acetyl-CoA production is a repressor of autophagy. This study also uncovered an interesting interconnection between different acetyl-CoA pools and their effects on autophagy. It was shown that blocking mitochondrial acetyl-CoA production by deleting the CoA-transferase *ACH1* caused an increase in the nucleocytosolic pool of acetyl-CoA. This was likely due to an accumulation of acetate, the substrate of acetyl-CoA synthetase Acs2, as well as a concomitant increase in *ACS2* expression. Increased acetyl-CoA results in repression of *ATG7* gene expression and an age-dependent defect in autophagic flux [112].

Interestingly, mammalian [190,192] and bacterial sirtuins [193] have been shown to regulate acetyl-CoA synthetase (ACS) activities. Mammalian SIRT1 and SIRT3 were shown to deacetylate and activate cytoplasmic ACS1 and mitochondrial ACS2, respectively [190]. CobB, a Sir2 ortholog in *Salmonella enterica* was shown to reactivate ACS, whose activity is almost completely inactivated by acetylation [193]. Most protein lysine acetylation studies in yeast have been centered on histone modifications mediated by HAT and HDAC. It remains unclear whether yeast sirtuins also directly regulate ACS activity. However, increased acetate utilization and acetyl-CoA production has been observed in cells lacking Sir2 [194]. The regulation of acetyl-CoA metabolism and histone deacetylation is reviewed in [195]. These findings demonstrate that acetyl-CoA levels may govern autophagy regulation through NAD^+^-dependent sirtuin activity and histone acetylation [112,113].

### 4.5. Copper Sensing

Copper is a critical trace element functioning in energy generation that can cause toxicity in high concentrations. Therefore, it must be tightly regulated. In a previous study, we identified the copper sensor Mac1 to be a novel NAD^+^ homeostasis factor [44]. Mac1-deficient mutants exhibit an increase in QA production, like that of Hst1-deficient mutants. Intracellular QA concentration was maintained at a low level in both strains while NAD(H) concentration increased after 6 h in media lacking NA. Due to the lack of NA, QA was converted to NAD^+^ and excess QA is excreted. Copper stress was shown to induce *BNA* gene expression, increasing QA production and release. However, copper stress was shown to slightly reduce NAD(H) levels. Hst1 and Mac1 mutant strains both result in the de-repression of the *BNA* genes, leading us to examine their promoter region. We identified that the *BNA2* gene, the rate-limiting step of the de novo pathway, lacks the Mac1 binding sequence in its promoter. Furthermore, Sum1 and Rfm1, which form a complex with Hst1, displayed increased QA release. Mac1 likely needs help from the Hst1-Sum1-Rfm1 complex to associate with the *BNA2* promoter, but the mechanism in which remains unclear. Interestingly, QA has been shown to form complexes with transition metal ions [196], leading us to speculate that QA may function as a copper chelator to facilitate its export under copper stress.

Increased concentrations of copper have been detected in the brains of patients with Alzheimer’s, Huntington’s, Parkinson’s, and Wilson’s disease [197]. Dysregulation of NAD^+^ metabolism and autophagy has been implicated in these diseases, suggesting a degree of interplay between copper sensing, NAD^+^ metabolism, and autophagy. Despite this, very little literature investigates the intersection between copper, NAD^+^, and autophagy. Exposure to excess copper has been shown to induce reproductive toxicity in men. Copper sulfate (CuSO_4_) administered to the mouse-derived spermatogonia cell line, GC-1, induced autophagy via the AMPK-mTOR axis [198]. The oxidative stress inhibitor N-acetylcysteine (NAC) attenuated CuSO_4_-induced autophagy, leading to an increase in ROS and a decline in cell viability. Excess mitochondrial copper induces adverse effects on the electron transport chain caused by increased ROS generation [199]. Mitophagy has been reported to ameliorate copper-induced mitochondrial dysfunction [199]. NAD^+^ dysregulation was not investigated in this study but, considering these effects, NAD^+^ metabolism may be altered in this model. Notably, an increase in NAD^+^ can trigger mitophagy in a sirtuin-dependent manner [200].

## 5. Selective Autophagy, Mitophagy, and NAD^+^ Metabolism

Mechanisms of mitophagy were discussed in Section 3.2. In yeast, mitophagy has been shown to be regulated by TORC1. Inhibition of TORC1 by the Seh1-associated complex inhibiting TORC1 (SEACIT) appears to stabilize the Atg32-Atg11 interaction, promoting mitophagy [149]. At the transcriptional level, the yeast Ume6-Sin3-Rpd3 complex represses *ATG32* gene transcription in cultures grown with a fermentable carbon source where the number of mitochondria and the rate of mitophagy is low [80]. This repression has been reported to be released by the inhibition of TOR by rapamycin [80]. Dep1, a component of this complex, is also critical for *ATG32* transcription and mitophagy [80]. NatA, an N-terminal acetyltransferase, appears to be required for the transcription of *ATG32* during nitrogen starvation where deletion of NatA causes mitophagy defects [201]. NatA contributes to Atg32 phosphorylation to promote interactions with Atg11, facilitating mitophagy [201]. The exact mechanisms of NatA’s involvement in mitophagy remain to be elucidated, but it is hypothesized that NatA acetylates an unidentified target that induces phosphorylation on Atg32. Similarly, it has been shown in yeast studies that NatB plays a crucial role in the regulation of the actin cytoskeleton and mitochondrial inheritance [116,117,118]. Reported NatB mutant defects were previously attributed to defective Nt-Ac of Tpm1/2. It was shown that cells with dysfunctional Mdm20 (the auxiliary subunit of NatB), or Nat3 (catalytic subunit of NatB) results in defects in actin cable formation and improper actin cable integrity, due to a lack of Tpm1 acetylation [118]. Interestingly, it was shown that the overexpression of Tpm1 (Tpm1-oe) and a gain-of-function Tpm1 mutant (Tpm1-5) could compensate for the NatB mutant-associated mitochondrial defects [116,117,118].

Moreover, mitophagy is crucial in mitochondrial quality control and regulation of ROS generation. Tissues lacking autophagy exhibit an accumulation of abnormal mitochondria [202]. Autophagy is tightly regulated by oxidative stress [203]. For example, ROS can directly oxidize the cysteine residues on ATG4, inactivating them, in mammals [204]. This affects autophagosome formation through a decrease in ATG4 activity, suppressing LC3 which is required for autophagosome expansion [205]. Likewise, suppression of mTOR by ROS has been shown to stimulate autophagy [206]. The antioxidant, N-acetylcysteine (NAC), has been shown to inhibit mitophagy by blocking Atg32 protein expression [207]. Autophagy-deficient cells exhibit elevated levels of ROS, which is exacerbated by nitrogen starvation [208]. Under nutrient rich conditions, mitophagy functions to selectively degrade damaged and superfluous mitochondria to maintain homeostasis [209].

NAD^+^ metabolism has been linked to mitophagy through several mechanisms. As discussed previously, the loss of mitochondrial quality control hyperactivates PARPs and sirtuins, leading to a decrease in the NAD^+^ pool [19]. PARP1 activation may also impair mitochondrial dysfunction and induce ATP depletion through glycolysis deactivation and decreasing NAD^+^ content [210]. PARP1 hyperactivation caused by unresolved DNA damage results in a loss in sirtuin activity inducing autophagy and, by extension, mitophagy abnormalities [211]. In yeast, Sir2 is required for the proper segregation of damaged proteins to the mother cell during budding [212,213]. Sir2 also regulates actin cables that are responsible for proper mitochondrial distribution [214]. Cells lacking Sir2 exhibit defects in these actin cables, resulting in a decrease in mitochondria quality [214]. Similarly, cells lacking Sir2 also exhibit increased sensitivity to ROS in the post-diauxic phase, further implicating NAD^+^ in the maintenance of healthy mitochondria [215]. The exhaustion of NADH in mitochondria triggers membrane depolarization and apoptosis and the dysregulation of NAD^+^:NADH ratio can increase ROS production [19]. Mitophagy may also function as a trigger for mitochondrial biogenesis. The suppression of mitochondrial function depletes ATP, increasing the AMP:ATP ratio and activating AMPK [216]. AMPK phosphorylates PGC1-alpha, activating mitochondrial biosynthesis factors [217,218]. Mitophagy actives Transcription Factor EB (TFEB) which also activates PGC1-alpha expression [218]. AMPK translocated to the mitochondria where it promotes mitophagy by recruiting VPS34 and ATG16 complex [27]. Likewise, Snf1 (yeast AMPK) is translocated to mitochondria, alongside Mec1, Atg1, and Atg13, in response to glucose starvation [34]. Mec1 phosphorylation, mediated by Snf1, is required for glucose starvation-induced autophagy via Atg1 [34]. Interestingly, mitochondrial respiration is also essential for glucose starvation-induced autophagy, which is mediated by Snf1, Mec1, Atg1, and Atg13 [34]. Furthermore, a recent study demonstrated that mitochondrial fusion machinery is required for the phosphorylation of Mec1 by Snf1 which facilitates the recruitment of Atg1 to the PAS under glucose starvation [219].

The link between ROS accumulation, NAD^+^ depletion, compromised mitophagy, and certain disease pathologies is highlighted in a recent review [220]. In line with this, the tight regulation of mitophagy is crucial to maintain a healthy mitochondria population and strategies to maintain mitophagy at a healthy level in aged individuals may be beneficial [221]. This may be accomplished through modulation of the machinery that directly activates mitophagy, like PINK1 and PRKN (the gene that encodes Parkin), or through the activation of sirtuins via STACs (Sirtuin-Activating Compounds), like resveratrol and metformin [220]. The supplementation of NAD^+^ precursors, like NR and NMN, or inhibition of NAD^+^ consumers, like PARPs, may also modulate mitophagy [222,223].

## 6. NAD^+^ Precursor Supplementation and Its Impact on Sirtuins and Autophagy

NAD^+^ levels and Sir2/SIRT1 protein concentration decline with age, resulting in detrimental age-related pathologies. As discussed previously, in mammalian systems, nuclear SIRT1 is subjected to clearance by autophagy in senescent cells [114]. NAD^+^ dysregulation has been seen in various human afflictions, including neurodegenerative and metabolic disorders, many of which also exhibit dysregulated autophagy. As NAD^+^ cannot be directly transported into cells, administration of NAD^+^ precursors may be viable therapeutics. Given the interconnection of NAD^+^ to other metabolic pathways, it is crucial to evaluate and understand the effects of NAD^+^ precursor supplementation. Special consideration should be given to the precursor supplemented, the concentration supplemented, the duration of supplementation, the disease context, and any off-target effects.

### 6.1. NAD^+^ Precursor Supplementation in Yeast

In yeast, NAD^+^ precursor supplementation has primarily been studied in the context of replicative and chronological lifespan. Replicative lifespan (RLS) is measured by the number of times a mother cell can replicate before senescence. Conversely, chronological lifespan (CLS) refers to the duration of time a population of yeast cells can survive in a post-mitotic state. Both methods can be used to evaluate the dynamics underlying aging and often are associated with different mechanisms of lifespan extension [224]. RLS appears to be mediated by Sir2, which suppresses recombination events in ribosomal DNA (rDNA) [225,226]. Sir2 deletion mutants display reduced RLS while Sir2 overexpression mutants result in extended RLS [227]. NAD^+^ depletion in aged cells suppresses Sir2 activity, decreasing RLS [38]. Because of this, NAD^+^ precursor supplementation in the context of Sir2 activity and RLS has been studied more extensively (reviewed in [228,229]). For example, the high concentration of NA in standard yeast media is associated with increased NAD^+^ content [44], supporting Sir2-mediated silencing. However, this requires Pnc1 to clear any generated NAM that may inhibit Sir2, and NAD^+^ levels are not maintained after NA depletion [230]. Cells lacking Npt1, the enzyme responsible for NA assimilation, display the opposite phenotype, characterized by a reduction in NAD^+^, loss of Sir2 silencing, and a decrease in RLS [150]. Notably, loss of Npt1 does not significantly affect CLS [55].

NAM, on the other hand, is a potent inhibitor of Sir2. Therefore, NAM supplementation is associated with a loss of Sir2-mediated silencing and a decrease in RLS [231]. Overexpression of Pnc1, the enzyme that catalyzes the conversion of NAM to NA, results in increased RLS as NAM clearance alleviates Sir2 inhibition [230]. Supplementation of isonicotinamide (INAM), which acts as a competitive inhibitor of NAM, alleviating Sir2 inhibition and facilitating an increase in NAD^+^ and RLS [232]. Interestingly, calorie restriction has also been reported to extend RLS in a Pnc1 and Sir2-independent manner [233,234]. CLS appears to be mediated by Pck1, the enzyme that regulates gluconeogenesis and a target of Sir2 deacetylation [235]. In CLS extension, an increase in acetylated Pck1, resulting in induction of gluconeogenesis, is favorable [235]. As such, a decrease in Sir2 activity facilitates gluconeogenesis induction via Pck1. Despite this, Sir2 itself has no observable effect on CLS extension [236]. NAM supplementation may be used to inhibit Sir2 activity and extend CLS through the induction of gluconeogenesis [237].

NR supplementation restores the loss of Sir2 silencing in cells lacking Npt1, abrogating RLS defects and increasing NAD^+^ content [54]. Cells lacking Nrk1, Urh1, and Pnp1 have attenuated NR salvage and significantly decreased RLS, highlighting the importance of this pathway to RLS [54]. In support of this, Sir2 deletion mutants have been shown to exhibit enhanced flow to the NR branch [238]. NR salvage is the most flexible and economical of the three NAD^+^ biosynthetic pathways, owed to by its dynamic compartmentalization and low metabolic cost [239]. Notably, NR supplementation in yeast cultures remains stable, while NA supplementation results in a decrease in NAD^+^ content over time [54,229]. It is unknown whether precursor supplementation impacts autophagy in the context of RLS and CLS in yeast.

### 6.2. NAD^+^ Precursor Supplementation in Mammals

Aberrant NAD^+^ metabolism has been implicated in many age-related diseases and metabolic disorders, including Parkinson’s Disease and obesity [240]. Substantial work on NAD^+^ precursor supplementation to remedy NAD^+^ metabolism-related defects has been conducted in mammalian systems. Furthermore, dysregulated autophagy has been implicated in many age-related diseases and metabolic disorders and a growing body of literature posits that autophagy plays a key role in NAD^+^ precursor supplementation [6,241]. Human embryonic stem cells (hESCs) defective in autophagy exhibit a decline in ATP and NAD^+^ caused by excess NADase activity [242]. SIRTs and PARPs are hyperactivated in response to elevated DNA damage, consuming NAD^+^. NAD^+^ decline results in elevated ROS and mitochondrial fragmentation, eventually leading to mitochondrial depolarization and cell death [242]. L-tryptophan, NAM, NR, and NMN supplementation were reported to increase NAD(H) and restore cell viability with NR and NMN exhibiting the most significant increase [242].

Similarly, heart failure with preserved ejection fraction (HFpEF) has been associated with a decrease in cardiac NAD^+^ concentration, which is ameliorated by NAM supplementation [243]. NAM was reported to stimulate mitophagy in cardiac cells, restoring mitochondrial quality control mediated by a decrease in IGF1 signaling [243]. Severe malnutrition induces dysregulation in the tryptophan-kynurenine pathway, which impairs intestinal function and structure [244]. Mice fed a low protein diet exhibited this trend and NAM supplementation induced SIRT1-mediated mitophagy [244]. NAM supplementation decreased intestinal damage and restored barrier function. Inhibition of mTORC1 with rapamycin stimulated autophagy, improving nutrient absorption and barrier function [244]. In this study, NAD^+^, SIRT1, mTORC1, and autophagy appear to function in concert in response to NAM supplementation in mouse and organoid models [244]. Furthermore, NAD^+^ depletion by hyperactive SIRTs and PARPs caused mitochondrial membrane depolarization in autophagy-deficient mice fibroblasts, leading to cell death [19,245]. Supplementation of NAM and NR and the pharmacological inhibition of SIRTs and PARPs increased NAD^+^ content and rescued the viability of these cells [19,245].

NR is one of the most promising geroprotective compounds and has been classified as Generally Recognized as Safe by the FDA [228,229]. Interestingly, supplementation of NAD^+^ precursors has been shown to significantly increase mitochondrial NAD^+^ levels in mammalian systems as well as modulate mitophagy and mitochondrial biogenesis in mammalian systems [200,246]. Inadequate blood flow to the brain causes chronic cerebral hypoperfusion (CCH) which has been linked to Alzheimer’s disease and vascular dementia. Administration of NR over three months has been shown to alleviate cognitive decline in rats with CCH [247]. NR maintained neuronal mitochondrial integrity, increased blood and brain NAD^+^ levels, and improved brain oxygenation [247]. Interestingly, this model exhibited an increase in expression of BECLIN1, a homolog of yeast Atg6/Vps30, indicating an increase in autophagy. NR supplementation alleviated this, restoring normal levels of autophagy. This may suggest that low NAD^+^ triggers autophagy and/or mitophagy which is mitigated by a boost in NAD^+^ via NR administration. Rats with CCH also exhibited an increase in mitochondrial fission via Drp1, which was reduced upon NR treatment. Drp1 has also been shown to regulate mitophagy, indicating that mitophagy may be involved in neuronal mitochondrial dysfunction [247]. Similarly, consumption of excess sweeteners has been shown to lead to cognitive decline [248]. Mice fed with sucrose and aspartame exhibited an influx in neuronal injury accompanied by a decrease in Nissl bodies and NAD^+^ levels. NR supplementation alleviated injury and boosted NAD^+^ levels. Surprisingly, NR supplementation also increased the amount of Nissl bodies, suggesting that NR exerted a protective effect on neurons. Furthermore, mice fed with sucrose and aspartame exhibited an increase in autophagy that was remedied by NR treatment [248]. Current clinical data cites NR supplementation to be safe and well tolerated [228].

NMN, a derivative of NR, has also shown promising results. NMN supplementation appears to increase NAD^+^ content by improving mitochondrial function, suppressing ROS generation and DNA damage, and slowing senescence [249]. For example, experiments in mice have demonstrated that NMN supplementation may protect against age-associated decline by enhancing mitochondrial metabolism and extending lifespan [250]. NMN supplementation has shown beneficial effects in several disease models. Alzheimer’s disease (AD) is characterized by deposits of beta-amyloid plaques and neurofibrillary tau protein tangles in the brain, resulting in neuronal loss [251]. Oxidative stress is often observed early on in AD, triggering excess phosphorylation of tau proteins, exacerbating neurofibrillary protein tangles. NMN administered to AD mice ameliorated oxidative stress, neuronal damage, and cognitive impairment [251]. Autophagic flux was shown to be decreased in AD mice by examining the LC3II:LC3I ratio, indicating that autophagosome formation was inhibited. NMN administration promoted autophagy and the clearance of p-tau [251]. In a similar vein, long term (7 months) NMN supplementation was shown to alleviate side effects of a high fat diet in mice [252]. NMN supplementation increased NAD^+^ concentration in mice fed a high fat diet [252]. This resulted in a concomitant increase in SIRT1 protein in skeletal muscle and inguinal white adipose tissue, while a high fat diet alone decreased SIRT1 proteins in these tissues [252]. NMN-treated mice exhibited a decline in obesity, improved glucose and lipid metabolism, increased physical activity, and improved skeletal muscle function. High fat diet induced excess autophagy in various tissues, which was inhibited upon NMN supplementation [252]. The role of NMN in mammalian systems has been discussed further in [253]. It remains unclear if sirtuins are always impacted by NAD^+^ precursor supplementation and if alterations in autophagy depend on sirtuins.

## 7. Conclusions and Future Perspectives

Due to its ubiquitous nature, NAD^+^ is implicated in numerous cellular processes, as evidenced by various mutant studies and the effects of precursor supplementation. This interconnection permits the tight regulation of NAD^+^ metabolism and nutrient sensing pathways like TOR, PKA and *PHO* signaling. Moreover, studies on NAD^+^ precursor supplementation have shed light on the potential mutual regulation of NAD^+^ metabolism and autophagy, especially under nutrient-limiting conditions.

Protein acetylation also plays an important role in regulating NAD^+^ metabolism and autophagy. The NatB complex directly links N-terminal acetylation to both autophagy regulation [115] and NAD^+^ homeostasis [13,72] through its essential role in the proper assembly of autophagosome formation machinery [115,116,117,118] and stabilization of key NAD^+^ biosynthetic enzymes, Nma1 and Nma2 [72]. Beyond protein-level acetylation modifications, the acetyl-CoA connection emphasizes the metabolic control of autophagy, where nucleocytosolic acetyl-CoA depletion promotes autophagy through reduced histone acetylation at *ATG* gene promoters [112]. In yeast, this mechanism involves the coordinated action of HATs and HDACs, including NAD^+^-dependent sirtuins, demonstrating that nucleocytosolic acetyl-CoA production may be a repressor of autophagy.

In mammals, deacetylation of autophagy-related proteins, like LC3 and ATG7, by sirtuins is regarded as a central inducer of autophagy, but this remains to be observed in *S. cerevisiae* [129]. Rather, yeast sirtuins appear to function in the transcriptional regulation of autophagy-related genes [132]. Given the conservation of sirtuins and autophagic mechanisms from yeast to humans, it may be warranted to investigate whether sirtuins mediate deacetylation of Atg proteins in *S. cerevisiae*, with emphasis on whether these interactions promote or inhibit autophagy. In line with this, the role of NAD^+^ precursor supplementation on autophagy remains to be fully understood. NAM supplementation appears to exert varying effects on autophagy, depending on the model systems and tissues profiled [19,230,232,237,243,245]. This may also depend on the concentrations of NAM supplemented. Low levels of NAM stimulate NAD^+^ biosynthesis, promoting sirtuin activity, but higher concentrations may inhibit the sirtuins regulating autophagy. The effect of NAM supplementation likely depends on the relative importance of sirtuin activity and NAD^+^ metabolism in the targeted process. The extent to which autophagy modulates NAD^+^ metabolism and the precursor pools is unknown. Autophagy appears to respond to nutrient starvation to varying degrees, depending on the nutrients and the extent to which they are deprived. In yeast, nitrogen starvation elicits a stronger autophagic response than glucose starvation [14], leading us to postulate whether low NAD^+^ conditions alone can stimulate autophagy.

The depletion of NAD^+^ content in mitochondria has severe implications for cellular health in cell lines deficient in autophagy. The loss of mitochondrial quality control results in dysregulated energy metabolism and DNA damage, which may hyperactivate sirtuins and PARPs, contributing to NAD^+^ decline [19]. These effects illustrate the interconnection between acetyl-CoA metabolism, NAD^+^ homeostasis, and autophagy, where mitochondrial NAD^+^ depletion triggers widespread metabolic dysfunction. Disruption of mitochondrial acetyl-CoA production can lead to compensatory increases in nucleocytosolic acetyl-CoA levels [112], which may affect autophagy through enhanced histone acetylation at *ATG* gene promoters. Further disentangling of the regulation of autophagy and NAD^+^ metabolism, especially in the context of their interconnection with nutrient signaling and acetylation dynamics, is crucial to understanding the effects of precursor supplementation. These studies may help advance the development of therapeutic strategies for diseases associated with age-dependent dysregulation of autophagy and NAD^+^ metabolism.

## Data Availability

Not applicable.
